# Gangrenous Cystitis Secondary to a Spontaneous Hematoma of the Lesser Pelvis

**DOI:** 10.7759/cureus.45502

**Published:** 2023-09-18

**Authors:** Panagiotis Angelopoulos, Stamatios Katsimperis, Ioannis Manolitsis, Themistoklis T Bellos, Lazaros Tzelves, Marinos Berdempes, Andreas Skolarikos

**Affiliations:** 1 Second Department of Urology, National and Kapodistrian University of Athens, Sismanogleio General Hospital, Athens, GRC

**Keywords:** radical cystectomy, acute abdomen, cystitis, partial cystectomy, gangrenous cystitis

## Abstract

Gangrenous cystitis is considered a life-threatening but rather rare clinical entity due to the widespread use of antibiotics. We herein report a case of a 78-year-old female with gangrenous cystitis secondary to a spontaneous expanded hematoma of the lesser pelvis who underwent partial cystectomy followed by bilateral ureterostomies with no favorable outcome.

## Introduction

Gangrenous cystitis is an extremely uncommon condition. Since 1934, only 204 cases have been described in the literature [1]. In recent years, the use of antibiotics has sharply reduced the number of reported cases [2]. In the majority of instances, conservative interventions supplemented by targeted antibiotic regimens have demonstrated sufficient efficacy. However, in cases marked by advanced clinical presentations, recourse to invasive interventions becomes imperative [2,3]. We hereby present a documented case wherein a patient underwent partial cystectomy to address the condition of gangrenous cystitis caused by microbial pathogens. Regrettably, the patient eventually succumbed. We present a brief review of the literature regarding the etiology, the diagnosis, and the management of this much-overlooked life-threatening infection.

## Case presentation

**Figure 1:**
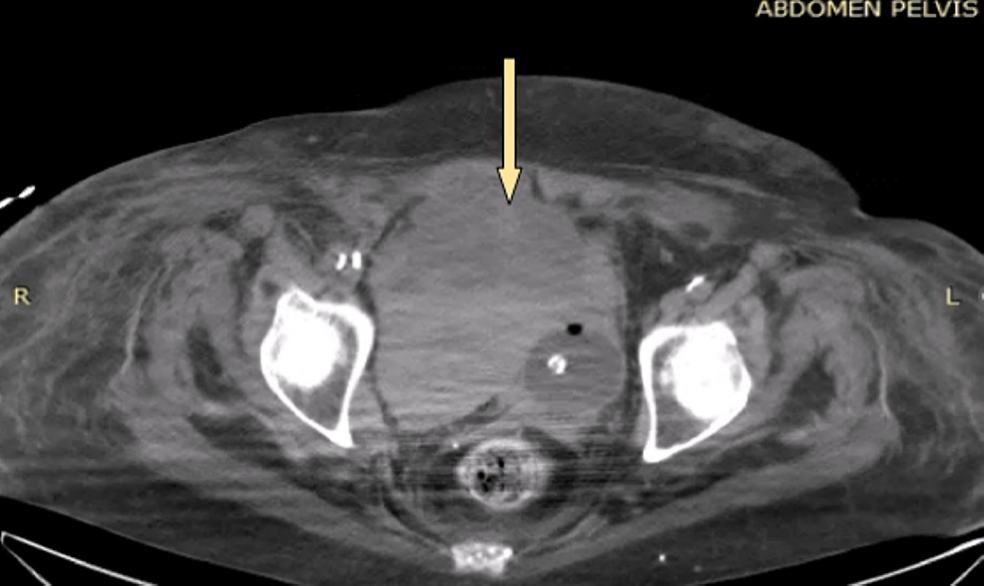
Large extraperitoneal hematoma in the lesser pelvis displacing the urinary bladder to the left (axial)

**Figure 2:**
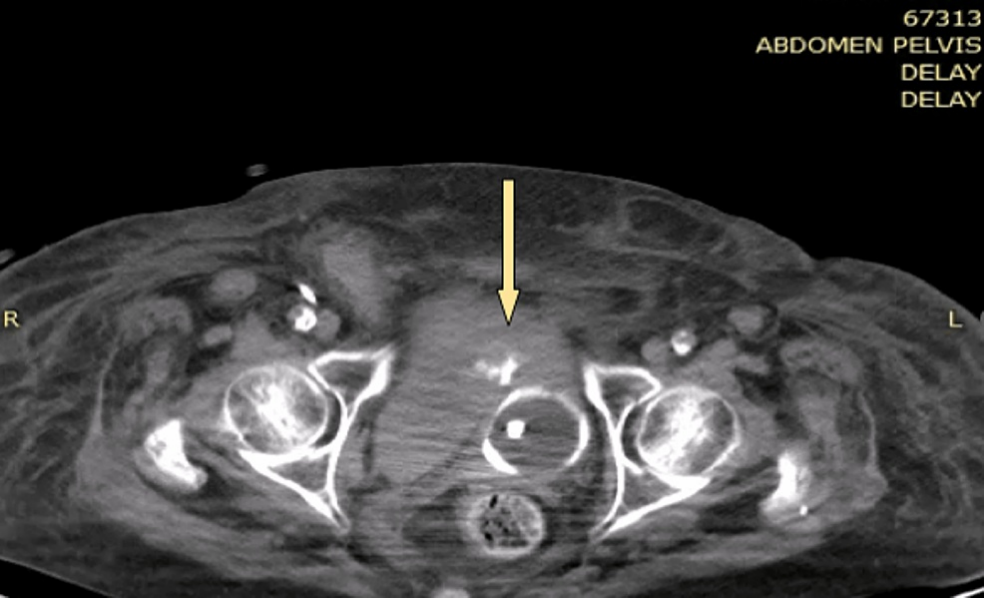
CT cystography showing contrast extravasation into the prevesical space (axial) CT: computed tomography

A 78-year-old patient, a nonsmoker, with a history of coronary artery disease, chronic obstructive pulmonary disease, and arterial hypertension, presented to the emergency department due to dyspnea. The urine culture was negative, as well as the screening for fungi. The patient was admitted to the pulmonary clinic, and having experienced an episode of atrial fibrillation, treatment with low-molecular-weight heparin was initiated. The patient's clinical condition gradually worsened, and ultimately, she was intubated and transferred to the intensive care unit (ICU). During her hospitalization in the ICU, the patient had an episode of macroscopic hematuria, a catheter was placed, and irrigations were performed, but the hematuria persisted, and the patient's clinical condition remained unstable. Therefore, she underwent a computed tomography (CT) scan, which revealed a large extraperitoneal hematoma in the lesser pelvis (Figure 1). A CT cystography followed, revealing contrast extravasation into the prevesical space (Figure 2). The patient consequently underwent an exploratory laparotomy for the removal of the hematoma and the suturing of the bladder rupture, while meticulous hemostasis was achieved without evidence of active bleeding.

**Figure 3:**
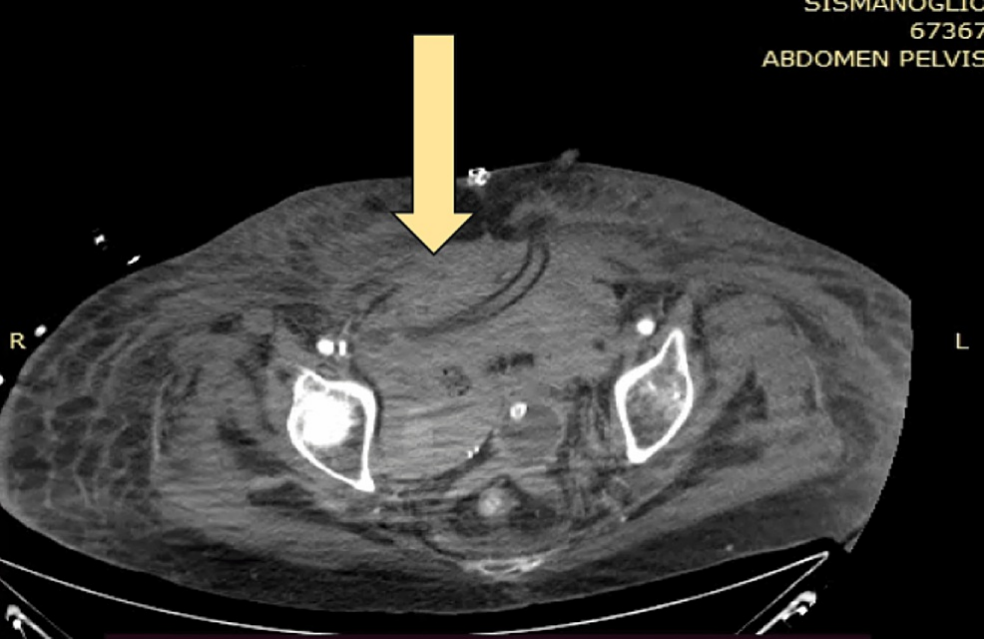
CT scan showing hematoma after the exploratory laparotomy (axial) CT: computed tomography

The patient on the 10th postoperative day presented with a worsened clinical status and was febrile, and a new CT scan showed a re-expanded hematoma in the lesser pelvis (Figure 3), while an increase in drainage fluids was noticed. A sample was collected and identified as urine.

**Figure 4:**
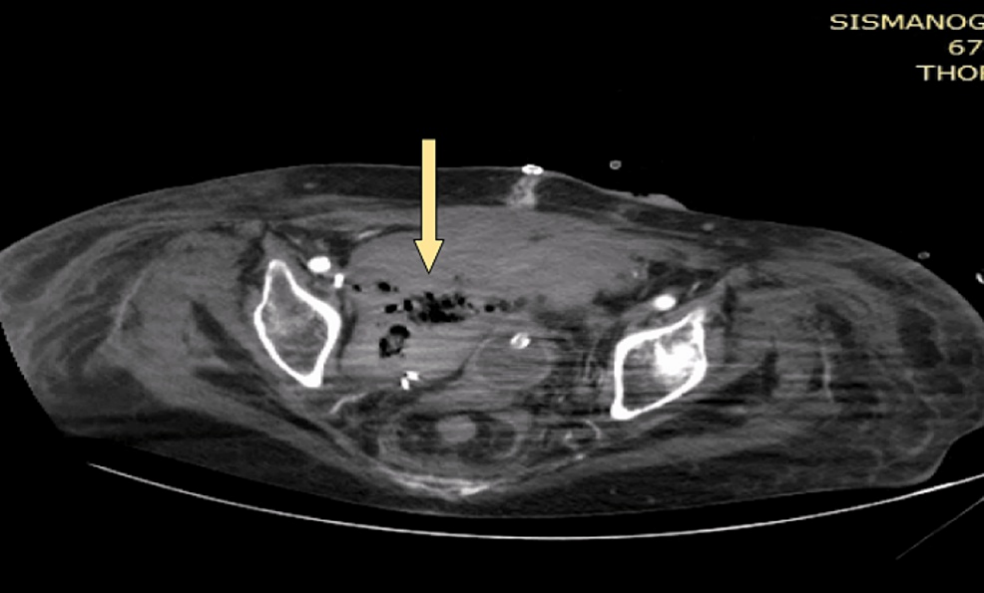
CT scan showing hematoma with air bubbles (axial) CT: computed tomography

Following the deterioration of the patient's clinical condition, a new computed tomography scan revealed the purulence of the hematoma with the presence of air bubbles (Figure 4), possibly due to gas-forming infection. A culture of pus obtained from the wound yielded three kinds of bacteria: *Pseudomonas aeruginosa*, *Enterococcus faecium*, and *Klebsiella pneumoniae*. The antibiotic treatment was upgraded to tigecycline and colistin from the initial regimen of meropenem and linezolid. The laboratory tests showed a noticeable worsening in their values (Table 1).

**Table 1:** Laboratory values

	The 10th postoperative day of exploratory laparotomy	Before the partial cystectomy	Normal range
White blood count	10.420/μL	16.700/μL	4.000-11.000/μL
Neutrophils	84%	91%	40%-75%
Hemoglobin	8.3 g/dL	7.8 g/dL	12-15 g/dL
Hematocrit	26.4%	23.6%	37%-45.6%
Platelets	271.000/μL	147.000/μL	150.000-400.000/μL
Urea	83 mg/dL	97 mg/dL	10-50 mg/dL
Creatinine	0.7 mg/dL	1.3 mg/dL	0.5-1.5 mg/dL
C-reactive protein	55 U/L	205 U/L	<6 U/L

The patient underwent a cystoscopy, revealing a significant deficit of the urinary bladder wall. The ureteral orifices were catheterized with double J stents. An exploratory laparotomy followed, necrotic tissue was found, and the area of the bladder triangle was examined without any pathological findings. It was decided to preserve it, and the necrotic area was removed. Partial cystectomy with bilateral ureterostomies was performed. Unfortunately, the patient succumbed after two days in the ICU. The report of the pathological autopsy described extensive lesions of ulcerative cystitis, with areas of interstitial hemorrhage and abscessed necrosis.

## Discussion

In the presented case, the most likely cause of bladder necrosis is the infection. The etiological factors might be classified as indirect and direct causes [2-4]. Indirect factors encompass elements that disrupt blood supply, giving rise to vascular complications and bladder ischemia. These factors are internal pressure (caused by prolonged urine retention and subsequent bladder overdistension), external pressure (stemming from conditions such as mispositioned pregnant uterus, extended labor, pelvic tumors, or surgical interventions), and the obstruction of significant arterial and venous pathways (resulting from ligation, embolism, or thrombophlebitis). Direct factors refer to those that inflict direct harm upon the bladder wall, culminating in cellular demise. These encompass a range of substances introduced into the bladder, pelvic radiation exposure, or extensive infection. This infection may arise from systemic sources (such as typhoid or diphtheria) or localized sources [5,6]. The isolated microorganisms span both aerobic and anaerobic varieties. Notably observed in individuals with diabetes, certain gas-producing bacteria can cause emphysematous cystitis, ultimately leading to complete bladder necrosis. Most of the patients resent comorbidities such as neurological conditions or diabetes mellitus [2].

The diagnosis is extremely difficult as the symptoms are not disease-specific. In most cases, the patient presents with dysuria, pyuria, hematuria, and even urinary retention with pain in the lower abdomen [4,7-9]. The condition of acute abdomen manifests as the patient presents in a state of advanced progression of the disease and is commonly concomitant with an unfavorable prognosis.

While computed tomography (CT) is the most useful diagnostic tool, it is typically requested when the patient's clinical presentation indicates a significant severity of symptoms (acute abdomen) [4,6,7]. Cystoscopy in particular is the only examination that permits a correct diagnosis to establish and may allow the debridement of necrotic tissue for culture [5].

The conservative approach, involving appropriate antibiotic therapy and the sealing of the urinary bladder, is often effective [10]. Surgical intervention is required in more advanced cases, where the patient undergoes either a radical or partial cystectomy with the preservation of the trigone [2,11,12]. Fortunately, the trigone usually remains viable because some of its blood supply comes from ureteral and prostatic vessels, and consequently, total cystectomy and urinary diversion are rarely required [5,6]. Gangrenous cystitis is a condition with a poor prognosis and a reported mortality of approximately 35% [2].

Over the recent years, various surgical approaches for replacing a removed bladder have gained prominence. These approaches can be broadly categorized into three main techniques: non-continent urinary diversions, continent non-orthotopic urinary diversions, and orthotopic urinary diversions [13]. Urinary tract infections (UTIs) are highly prevalent and often linked to significant morbidity [14].

## Conclusions

The cases of gangrenous cystitis are still observed despite the widespread use of antibiotics. A clinical physician should maintain a high degree of suspicion in most cases to arrive at a diagnosis. Nevertheless, early diagnosis can often prove decisive for patients, as delayed diagnosis increases the mortality rate. Treatment in such cases is surgical.
